# A protocol for the emergency department management of acute undifferentiated febrile illness in India

**DOI:** 10.1186/1865-1380-4-57

**Published:** 2011-09-05

**Authors:** Sudhagar Thangarasu, Piruthiviraj Natarajan, Parivalavan Rajavelu, Arjun Rajagopalan, Jeremy S Seelinger Devey

**Affiliations:** 1 Dept. of Internal Medicine, University of Pittsburgh Medical Center-Mercy Hospital, 1400 Locust Street, Pittsburgh, PA 15206, USA; 2 Dept. of Emergency Medicine, Sundaram Medical Foundation, Shanthi Colony, 4th Avenue, Anna Nagar, Chennai - 600040, India; 3 Dept. of Surgery, Sundaram Medical Foundation, Shanthi Colony, 4th Avenue, Anna Nagar, Chennai - 600040, India; 4 Dept. of International Emergency Medicine, Long Island Jewish Medical Center, 270-05 76th Ave., New Hyde Park, NY 11040, USA

## Abstract

**Background:**

Fever is a common presenting complaint in the developing world, but there is a paucity of literature to guide investigation and treatment of the adult patient presenting with fever and no localizing symptoms.

**Objective:**

The objective of this study was to devise a standardized protocol for the evaluation and treatment of febrile adult patients who have no localizing symptoms in order to reduce unnecessary testing and inappropriate antimicrobial use. After devising the protocol, a pilot study was performed to assess its feasibility in the emergency department.

**Methods:**

A protocol was formulated for adult patients presenting with fever who had no clinical evidence of sepsis and no localizing symptoms to suggest the etiology of their fever. Investigations were based on duration of fever with no investigations indicated prior to day 3. Treatment was guided by results of investigations. A pilot study was performed after protocol implementation, wherein data were collected on successive adult patients presenting with fever.

**Results:**

During the 6-week study period, 342 patients presented with fever, 209 of whom fit the parameters of the protocol, with 113 of these patients presenting on the 1st or 2nd day of fever. All patients experienced defervescence of fever, with ten patients being lost to follow-up. Of the patients presenting on day 1 or 2 of fever, 75.2% (85/113) defervesced without the need for testing; 53.1% (60/113) experienced defervescence without the need for antimicrobial therapy.

**Conclusion:**

Implementation of this rational, standardized protocol for the assessment and treatment of stable adult patients presenting with acute undifferentiated febrile illness can lead to reduced rates of testing and antimicrobial use. A prospective, controlled trial will be required to confirm these findings and to assess additional safety outcome measures.

## Introduction

Fever is a common presenting complaint in the developing world and is the most common presentation to the Emergency Department (ED) at our institution, Sundaram Medical Foundation (SMF) in Chennai, India [1]. Febrile illness can be localized to organ systems or non-localized, commonly referred to as acute undifferentiated febrile illness (AUFI). In the Western world, AUFI is often due to self-limited viral conditions. However, in the developing world, the differential diagnosis for AUFI includes potentially significant illnesses such as malaria, dengue fever, enteric fever, leptospirosis, rickettsiosis, hantavirus, and Japanese encephalitis [2-10]. There is a paucity of literature on the appropriate evaluation of adult fever patients without localizing symptoms in the ED [11]. In the absence of established protocols, patients may be subjected to unnecessary investigations at considerable cost and the inappropriate prescribing of antimicrobial therapy [12,13]. In the following, we describe a protocol that was formulated and implemented in the SMF ED to evaluate adult patients presenting with non-localizing fever.

## Objective

The aim of this pilot study was to devise and implement a protocol for the management of stable adult patients presenting to the emergency department with fever as their chief complaint and no localizing symptoms. The overarching goal of the protocol was to standardize the approach to such patients in a way that reduced unnecessary testing and inappropriate use of antibiotics. Additional goals, such as improving time to fever resolution, reduction in hospital admission rate, and reduction in mortality, while also ultimately desirable, were not assessed in this study.

## Methods

**Figure 1:**
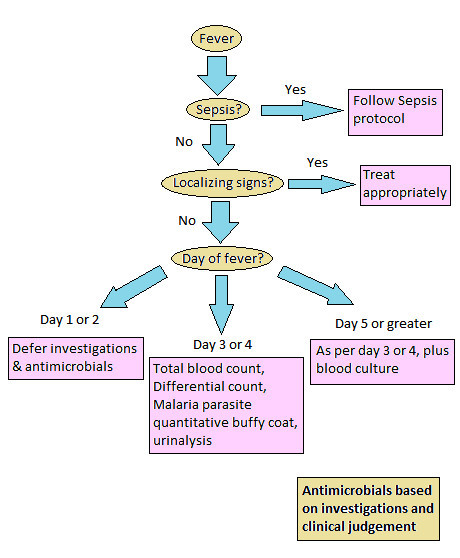
**Protocol for the management of adult patients with acute undifferentiated fever**.

A protocol for the management of stable adult patients presenting to the SMF ED with a chief complaint of fever was devised according to the local infectious epidemiology by SMF emergency physicians in consultation with SMF medicine consultants and is presented in Figure 1. All adult patients aged 17 and older with a presenting complaint of fever but without localizing symptoms were considered for evaluation by the protocol. Patients with localizing symptoms that suggested the etiology of fever and those meeting criteria for severe sepsis or septic shock were excluded. Eligible patients were managed either by the protocol or as deemed most appropriate by the evaluating physician. Under the protocol, if an eligible patient was stable and had had less than 3 days of fever, all investigations and antimicrobial therapy were deferred, and the patient was prescribed antipyretics and asked to return to the ED on the 3rd day of fever if it persisted. Patients presenting on days 3 or 4 of fever had total blood count, differential count, malaria parasite quantitative buffy coat test, and urinalysis performed. Patients presenting on day 5 or greater of fever additionally had a blood culture performed. All patients were then treated according to the results of investigations as deemed appropriate.

In order to assess the feasibility of the implementation of this protocol, data were prospectively collected on all eligible patients presenting to the SMF ED between 1 August 2008 and 15 September 2008. Data collected included day of fever at presentation, day of fever resolution, investigations performed, antimicrobial therapy received or not, and final diagnosis. Thirty-day follow-up was performed by phone interview and examination of medical records to assess final outcomes. The study protocol was reviewed and approved by the IRB at Sundaram Medical Foundation.

## Results

**Figure 2:**
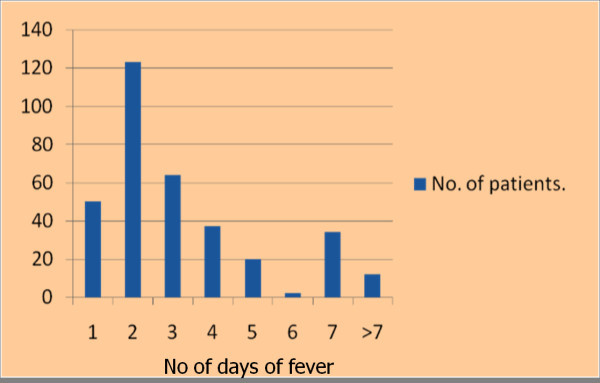
**Day of fever at the time of presentation**.

During the study period 342 patients presented with fever. Of these, 6 (1.8%) met the clinical definition of sepsis and were treated according to sepsis protocol, and 127 (37.1%) had localizing symptoms to suggest an etiology for their fever. This left 209 patients (61.1%) with AUFI eligible for the protocol. The majority of these patients were presenting on the 1st or 2nd day of fever (Figure 2).

Of the 113 AUFI patients who presented within the first 2 days of fever, 57.5% (65/113) were treated according to the protocol and received no investigations (Table 1). Of these, 75.4% (49/65) experienced spontaneous defervescence, while the remainder underwent investigation per the protocol at the 3- and 5-day follow-up. Among the 48 patients presenting within the first 2 days of fever who underwent investigations outside of the protocol, all experienced defervescence. The investigations were contributory to patient management in 25.0% (12/48) of these cases and did not change management in the remaining 75.0% (36/48). Four patients were lost to follow-up. Investigations were ultimately unnecessary in 75.2% of patients (49 who defervesced without investigation plus 36 who had non-contributory investigations and defervesced out of 113 patients) presenting on the 1st or 2nd day of fever.

**Table 1:** Outcomes of stable adult patients with acute undifferentiated febrile illness presenting on day 1 or 2 of fever

	Number	Percent*
Eligible patients, day 1 or 2 of fever	113	100%
Received investigations initially	48	42.5%
Investigations contributory	12	25%
Investigations non-contributory	36	75%
Did not receive investigations initially	65	57.5%
Defervesced without need for investigations	49	75.4%
Eventually investigated as per protocol	12	12.7%
Lost to follow-up	4	6.2%
**Total defervesced without need for investigations**	**85**	**75.2%**
Received antimicrobials initially	35	31%
Did not receive antimicrobials initially	78	69%
Defervesced without need for antimicrobials	60	87%
Eventually required antimicrobials	15	19.2%
Lost to follow-up	3	3.8%
**Total defervesced without need for antimicrobials**	**60**	**53.1%**

*Percentages calculated using subcategory as denominator.

**Bold **items highlighted to illustrate the potential for reduction in unnecessary investigations and inappropriate antimicrobial therapy.

Antimicrobial therapy was prescribed to 35 of the 113 AUFI patients who initially presented within the first 2 days of fever and ultimately received at a later date by 15 additional patients. Three patients were lost to follow-up. Of the patients, 53.1% (60/113) experienced defervescence without the need for antimicrobial therapy.

**Figure 3:**
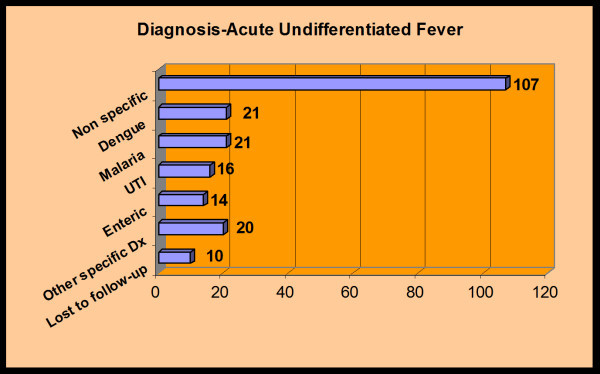
**Final diagnosis of adult patients with acute undifferentiated fever**.

All patients experienced resolution of fever, with ten being lost to follow-up. The final etiology of fever was never determined in the majority of cases (Figure 3).

## Discussion

Given the relative frequency with which emergency physicians in India encounter patients with acute undifferentiated febrile illness, it is in our interest to develop a standardized approach to evaluating these patients. Evidence-based protocols have been shown to be cost-effective [14] and improve mortality [15] in the emergency department setting. This protocol has the more modest goals of reducing costs, avoiding unnecessary testing and inappropriate therapies, and reducing antibiotic resistance and rates of misdiagnosis. We have described a protocol that represents a rational, graded approach to stable adult patients with AUFI that is informed by local infectious epidemiology [2]. In this pilot study, investigations were or could have been avoided in 75.2% of patients, and antimicrobial therapy was unnecessary for fever resolution in 53.1% of eligible patients with fever of < 3 days duration. These data suggest that this protocol has the potential to reduce unnecessary testing and inappropriate antimicrobial use. A prospective trial will need to be carried out both to corroborate these findings as well as to investigate the ability of the protocol to influence additional outcome measures such as time to fever resolution, hospital admission rate, and mortality rate.

## Conclusion

Implementation of a rational, standardized protocol for the assessment of stable adult patients with acute undifferentiated febrile illness in this south Indian emergency department demonstrates a potential to lower rates of unnecessary testing and antimicrobial use. The protocol will need to be prospectively validated in a controlled fashion in order to confirm these findings as well as to assess its safety.

## List of abbreviations

ED: Emergency department; SMF: Sundaram Medical Foundation, Chennai, Tamil Nadu, India; AUFI: acute undifferentiated febrile illness; IRB: institutional review board.

## Competing interests

The authors declare that they have no competing interests.

## Authors' contributions

TS designed the study and collected data; NP collected data and followed up patients, PVR designed the study, supervised data collection and edited manuscript; and AR supervised the study design and edited the manuscript. JSD reviewed the available literature, edited for content, and prepared the manuscript. All authors read and approved the final manuscript.

## Author's information

TS is a Resident Physician in Internal Medicine, University of Pittsburgh Medical Center-Mercy Hospital. NP is Senior House Officer in Emergency Medicine at Sundaram Medical Foundation. PVR is Head of Department, Department of Emergency Medicine at Sundaram Medical Foundation. AR is Medical Director and Head of Department, Department of Surgery at Sundaram Medical Foundation. JSD is International Emergency Medicine Fellow at Long Island Jewish Medical Center.
